# Eruptions of the Skin during Pregnancy

**Published:** 1851-05

**Authors:** Hiram Allen


### ARTICLE V.

Eruptions of the Skin during Pregnancy.—Read at the Quarterly Meeting of the Rhode Island Medical Society, by Dr. Hiram Allen, and communicated by the Publishing Committee.

Two cases of disease of the skin have recently occurred in my practice, which were different from any cases which I now recollect of having witnessed before. The first case was a woman who was between eight and nine months advanced in pregnancy, and had been quite well during the whole time until a few days before I saw her. She complained of an excessive itching of the skin over the system, attended with a fine eruption, differing in appearance from ordinary eruptions, The elevations of the surface were fine, distinct, of a light brownish color. I prescribed some mild applications to allay the itchings, but all were unavailing until her delivery, which revealed and removed the cause of her external disease. She was confined, as she supposed, a week before her time had expired, and was delivered of a stillborn child. From its appearance I should think the child had been dead three weeks; decomposition had begun to take place, and the skin had sloughed off in some places. From the symptoms, the mother supposed the child must have died about three weeks previous to its birth. The woman soon recovered, and all the eruption rapidly disappeared. The disease of the surface I suppose was the effect of nature's depurating process to rid the system of the intra-uterine poison, which had been absorbed.

I have had a similar case since. The woman had an itching and eruption of the skin, which appeared in many respects similar to the other case; but upon inquiry I found she felt active motion of the child. I concluded her child must be living, but the eruption indicated the reverse. At the time of birth I officiated. The woman had twins. One appeared to be well and healthy—the other, from its decomposed state, had probably been dead for several weeks.—*Boston Med. and Surg. Jour.*

